# NABNet: Deep Learning-Based IoT Alert System for Detection of Abnormal Neck Behavior

**DOI:** 10.3390/s24165379

**Published:** 2024-08-20

**Authors:** Hongshuai Qin, Minya Cai, Huibin Qin

**Affiliations:** School of Computer Science, Hangzhou Dianzi University, Hangzhou 310018, China

**Keywords:** abnormal behavior detection, pose estimation, YOLOv5s, lightweight model, IoT system

## Abstract

The excessive use of electronic devices for prolonged periods has led to problems such as neck pain and pressure injury in sedentary people. If not detected and corrected early, these issues can cause serious risks to physical health. Detectors for generic objects cannot adequately capture such subtle neck behaviors, resulting in missed detections. In this paper, we explore a deep learning-based solution for detecting abnormal behavior of the neck and propose a model called NABNet that combines object detection based on YOLOv5s with pose estimation based on Lightweight OpenPose. NABNet extracts the detailed behavior characteristics of the neck from global to local and detects abnormal behavior by analyzing the angle of the data. We deployed NABNet on the cloud and edge devices to achieve remote monitoring and abnormal behavior alarms. Finally, we applied the resulting NABNet-based IoT system for abnormal behavior detection in order to evaluate its effectiveness. The experimental results show that our system can effectively detect abnormal neck behavior and raise alarms on the cloud platform, with the highest accuracy reaching 94.13%.

## 1. Introduction

Sedentary lifestyles are common among young people today due to high dependence on electronic products such as mobile phones and computers. The incorrect sitting position is one of the manifestations of neck diseases [1,2]. Early detection and monitoring of abnormal behaviors can improve the life quality of sedentary people while reducing pressure on medical resources and providing significant commercial value.

Abnormal behavior can be defined as actions in which people may be in danger [3]. In this paper, we identify long-term head tilts and dropped heads as abnormal neck behaviors. Traditional methods rely on ambient devices to obtain environmental data and human data for abnormal behavior monitoring [4,5,6]. This approach is sensitive to environmental noise and lacks flexibility.

In recent years, studies about abnormal behavior detection have indicated that convolutional neural network (CNN)-based computer vision possesses high robustness and accuracy for detection [3,7,8]. While object detection-based methods are suitable for full-body abnormal behaviors such as falls and repeated activities [9,10], they are ineffective for detecting abnormal neck behavior, which involves smaller localized movements.

In this paper, we propose an intelligent detection and monitoring solution for abnormal neck behavior in indoor scenarios, called Neck Abnormal Behavior Network (NABNet). NABNet is designed to detect head tilt and dropped head events. First, NABNet uses YOLOv5s to extract features from the input in order to detect and track objects [11]. To improve the accuracy of small target detection, the Coordinate Attention (CA) [12] mechanism is added to YOLOv5s. Second, we obtain the key points and joint information of the body using Lightweight OpenPose [13]. Abnormal behavior events are detected by body orientation judgment, angle calculation, and correction. The high computational overhead of existing CNN-based models makes them challenging to execute on edge-end IoT devices while maintaining good real-time performance. Thus, we built our proposed NABNet IoT-based alert system to quickly and cheaply deployed on edge devices. Abnormal neck behaviors are detected in real-time, allowing alerts to be sent to healthcare professionals or caregivers.

Our main contributions can be summarized as follows:

1. This paper proposes a deep learning-based solution for monitoring and detecting abnormal neck behavior in sedentary people; specifically, the proposed NABNet detects head tilt and dropped head events.
2. The features inherent in abnormal neck behaviors are fully considered to alleviate false alarms. NABNet combines YOLOv5s with a CA mechanism to enhance the robustness of object detection, then uses OpenPose-guided skeleton and angle relationship information to judge the neck position.
3. A NABNet-based detection system was deployed on edge-end IoT devices, and its performance was tested in practical scenarios. Our experimental results demonstrate the effectiveness of NABNet for detecting abnormal neck behavior.

## 2. Related Works

### 2.1. Object Detection

In recent years, object detection methods based on neural network algorithms have become widely used in industrial fields, in particular for intelligent monitoring systems [14,15]. Current mainstream object detection algorithms are mainly categorized into two-stage and single-stage approaches [3]. The former approach generates a series of samples of the candidate box and then uses a CNN for sample classification. Two-stage approaches include networks such as the R-CNN [16], faster R-CNN [17], R-FCN [18], and Libra R-CNN [19]. The latter approach does not have to generate a candidate box, instead directly transforming the problem of target box localization into a regression problem [20]. Single-stage approaches include networks such as YOLO [21], SSD [22], and RetinaNet [23]. Single-stage algorithms are superior in terms of speed and have increasingly been applied in practical detection applications, including medical cancer cell detection [24], face detection [25], product detection [26], and more. However, their detection accuracy is slightly lower than two-stage algorithms, and they are not good at detecting small objects [27].

Thus, these state-of-the-art object detectors are ineffective for detecting abnormal neck behavior, as they cannot capture subtle feature differences such as neck rotation, which are critical for the classification and localization of abnormal neck behaviors. In this paper, the neck behavior features are learned by combining object detection and pose estimation to obtain better abnormal behavior detection results.

### 2.2. Abnormal Behavior Detection

Current methods for abnormal behavior detection can be coarsely classified into three types: ambient device-based, wearable-based, and computer vision-based [28,29]. The ambient device-based [30,31] and wearable sensor-based [28,32] methods require dedicated devices to collect ambient data (e.g., floor pressure, sound, vibration) or object movement data (e.g., speed, acceleration). However, ambient device-based methods are sensitive to environmental noise, leading to high false positive rates [29]. Ma, C. et al. [4] used pressure sensors to detect abnormal behaviors of people in wheelchairs, using a fuzzy inference system to evaluate movement and posture conversion intensity. Based on pressure sensors, PIR sensors, etc., Arifoglu, D. et al. [5] identified abnormal behaviors such as forgetting or repetition in the elderly. Tokas, P. et al. [6] used a Microsoft Kinect sensor to detect normal and abnormal sitting postures, achieving the highest accuracy of 92.85% with Random Forest and Support Vector Machine classifiers. Wearable-based approaches require individuals to wear sensors, which can cause discomfort and inconvenience. On the other hand, computer vision-based approaches usually use cameras to capture images, overcoming the limitations of fixed device placement inherent in wearable-based methods and allowing for simultaneous and visible capture of multiple events.

Many image-based abnormal behavior recognition works have emerged thanks to the popularity of camera monitoring and the powerful feature extraction ability of deep learning. The core technology of abnormal behavior detection is to recognize the category and location of abnormal behaviors in images captured by camera monitoring [3]. An increasing number of studies have been emerging into abnormal behavior detection as a subtask of object detection. Alruwaili, M. et al. [33] proposed a Yolov5-based real-time detection and tracking model for people with paralysis, limb defects, and other diseases. Wang L. et al. [2] proposed an attention-based spatiotemporal network to identify the behavior and location of abnormal activities in the elderly, such as multiple toilet visits, forgetting to wash dishes, etc. Fang, M.-T. et al. [34] proposed a real-time abnormal behavior detection method using improved YOLOv3. These methods attempt to handle abnormal behavior detection through improved generic object detection methods based on deep learning and temporal surveillance information.

In addition, many methods try to define different kinds of abnormal behavior in advance depending on the specific scenario. Mehmood, A. et al. [35] defined human falls, certain types of suspicious behavior, and violent acts as abnormal activities. They provided a lightweight framework to represent and differentiate between normal and abnormal events effectively. Fu, Y. et al. [8] proposed a lightweight GD-YOLO network based on YOLOv7 for detecting abnormal behaviors, including smoking and using mobile phones.

In this paper, based on behavior in office scenarios, we define head tilt and dropped head postures as abnormal behaviors of the neck. We focus on detecting abnormal behavior of the neck joints and combine object detection with pose estimation to better detect abnormal behavior.

## 3. Methods

### 3.1. Overview

**Figure 1:**
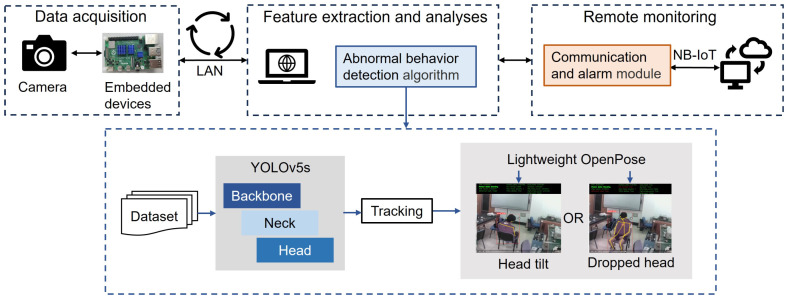
Schematic of the proposed NABNet-based IoT system for abnormal behavior detection.

This paper proposes an abnormal behavior detection network called NABNet based on YOLOv5s and Lightweight OpenPose for detecting head tilt postures of sedentary people in indoor scenarios. Furthermore, we present a NABNet-based IoT system that leverages the trained NABNet and hardware equipment, as shown in Figure 1. Video is collected by a camera and processed on edge devices to detect abnormal behaviors, with the results uploaded to the cloud for issuing alarms. The system’s main components are video gathering, image transmission and processing, and NABNet-based detection parts. Based on the YOLOv5s object detection network and the Lightweight OpenPose pose estimation network, NABNet extracts the detailed behavior characteristics of the head and neck from global to local to detect abnormal head tilt and dropped head behaviors.

### 3.2. Object Detection and Tracking

**Figure 2:**
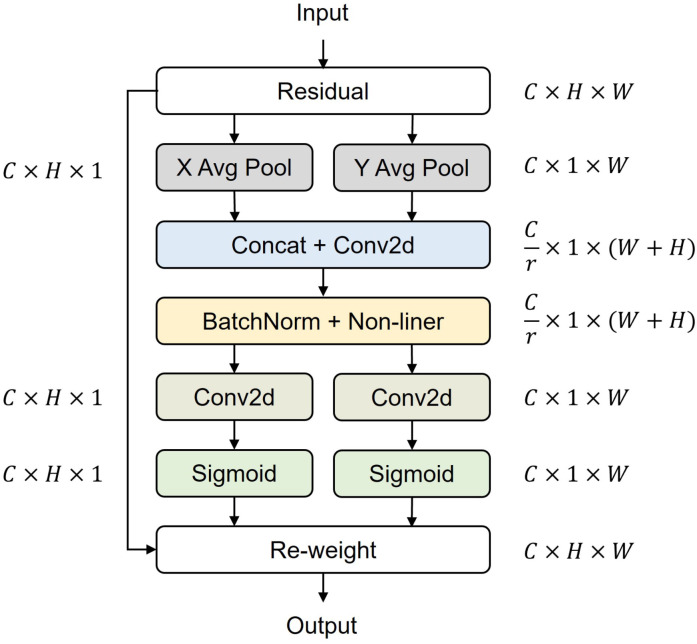
CA mechanism structure.

YOLOv5s is a CNN-based object detection model composed of backbone, neck, and head networks. The input is processed by the backbone network (CSPDarknet53 [36]) for feature extraction, the neck network for feature fusion, and the head network for object prediction.The backbone network uses Focus, CBL, CSP, and SPP structures to reduce the calculation without reducing the accuracy [37]. The neck network uses top-down FPN and bottom-up PAN modules for feature fusion. Finally, we adopt the GIOU loss to supervise the network [38]. The CA mechanism is embedded in the backbone structure of YOLOv5s to enhance the feature extraction and object attention capability, as shown in Figure 2. Unlike other mechanisms that convert the features extracted by the network into a single feature vector through 2D global pooling, the CA mechanism decomposes channel attention into two 1D feature encoding processes and then separately aggregates features along two spatial directions; one spatial direction captures long-range dependencies, while the other retains precise positional information. Two feature maps with specific spatial dependencies are obtained by applying an activation function, enhancing attention towards the target of interest.

**Figure 3:**
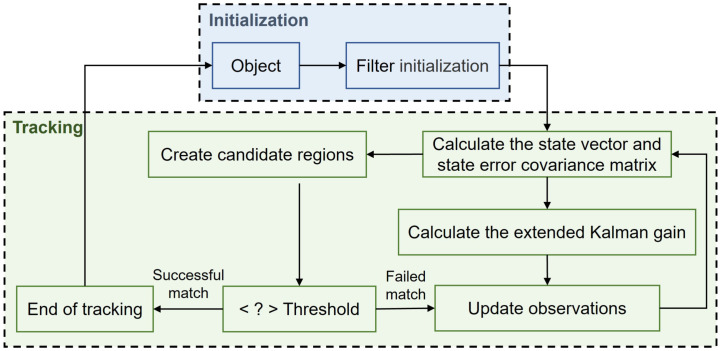
Flowchart of object tracking.

We track the object to analyze the behavior category according to the object’s motion mode. For this, we design an extended Kalman filter-based tracker, as shown in Figure 3. The tracking process is divided into two parts: initialization and tracking; in this paper, the frame of YOLOv5s detection failure is taken as the third frame of the tracker, while the first two frames are taken as the tracker’s initial state. After initialization, the prior state and error covariance matrix are calculated, and multiple candidate regions are created near the preceding state. The candidate region is matched with the tracking result of the previous frame using the difference hash to select the best-matching candidate region. The matching value is compared with a threshold. If this threshold is exceeded, the best-matching candidate region is used as the observation value to update the system; otherwise, the matching is considered to have failed, and waiting for the detector to detect the object again is necessary.

The traditional Kalman filter assumes a Gaussian distribution and linearity; however, object tracking of the body is nonlinear due to object deformation and environmental changes. The extended Kalman filter can solve the problem of nonlinearity via Taylor expansion [39]. The state equation and observation equation of the extended Kalman filter are as follows:

(1) $$x_{k}=f\left(x_{k-1}\right)+w_{k-1},$$

(2) $$z_{k}=h\left(x_{k}\right)+v_{k},$$

where $x_{k}$ and $x_{k-1}$ denote the state vector with *k* and k−1, respectively, f() is the state transition function, and $w_{k-1}$ is the process noise, which is assumed to conform to the multivariate normal distribution with a mean value of 0 and covariance matrix of Q. The process noise is due to uncertain factors in practical scenarios, such as sudden acceleration, deceleration, and turning. In addition, $z_{k}$ is the observation vector, h() is the state observation function which converts the state space into the observation space, and $v_{k}$ is the observation noise, which is assumed to conform to a multivariate normal distribution with a mean value of 0 and covariance matrix of R.

### 3.3. Detection of Abnormal Neck Behavior

Because the neck has subtle movements, using the object detection method directly is impossible, as it can only make judgments based on violent movements (see Section 3.2). Inspired by Maji D. et al. [40], this paper uses the YOLOv5s object detection model combined with the Lightweight OpenPose pose estimation method [13,41] to determine the neck’s state. The advantages of this approach are as follows: (1) combining YOLOv5s with Lightweight OpenPose increases the robustness of the model against occlusion compared to using Lightweight OpenPose alone, which can cause detection errors if key points and joint information are lost; (2) having Lightweight OpenPose only process the objects extracted by YOLOv5s helps it to ignore irrelevant objects, which reduces the amount of calculation and improves real-time performance.

**Figure 4:**
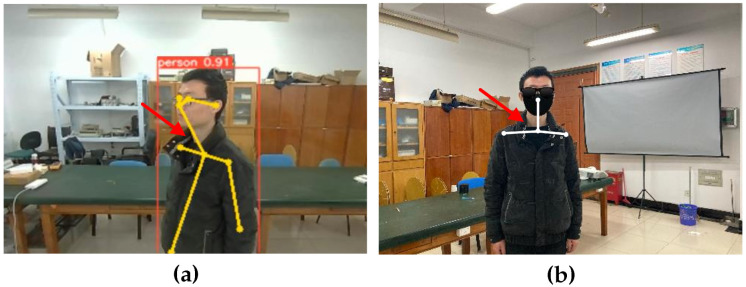
(**a**) The neck state when the object side is towards the camera and (**b**) the actual neck state.

**Figure 5:**
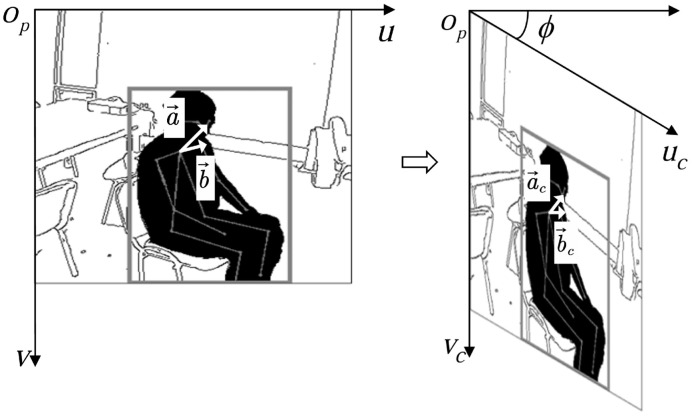
Angle correction via affine transformation. $\vec{a}$ denotes the vector between the neck and the head, and $\vec{b}$ represents the vector between the neck and the shoulder.

We determine abnormal behavior according to the neck angle. The evaluation criteria of the dropped head state consist of the ratio of the neck-to-nose vector to the vector from the neck to the shoulder on one side. The evaluation criteria of the head tilt state are the angle between the neck and the shoulder. When the camera is not in the front position of the object, there is a difference between the detected angle and the actual angle of the neck, as shown in Figure 4. To solve this problem, we first determine the body’s orientation, then use the camera calibration to obtain the mapping relationship between the two-dimensional and three-dimensional object, and finally obtain the angle between the object and the camera by calculating the Euler angle. Then, the angle correction is carried out by affine transformation [42] to reduce the timidity of the angles, as shown in Figure 5. The affine transformation is as follows:

(3) $$\left(\begin{array}{c} u_{c}\\ v_{c}\\ 1 \end{array}\right)=\left(\begin{array}{ccc} \cos\phi & 0 & 0\\ -\sin\phi & 1 & 0\\ 0 & 0 & 1 \end{array}\right)\left(\begin{array}{c} u\\ v\\ 1 \end{array}\right)$$

where ϕ is the yaw angle and *w* is the neck angle, determined as follows:

(4) $$w=arc\cos(\frac{\vec{a_{c}}\cdot \vec{b_{c}}}{∥\vec{a_{c}}∥∥\vec{b_{c}}∥}).$$

## 4. NABNet-Based IoT Alert System

**Figure 6:**
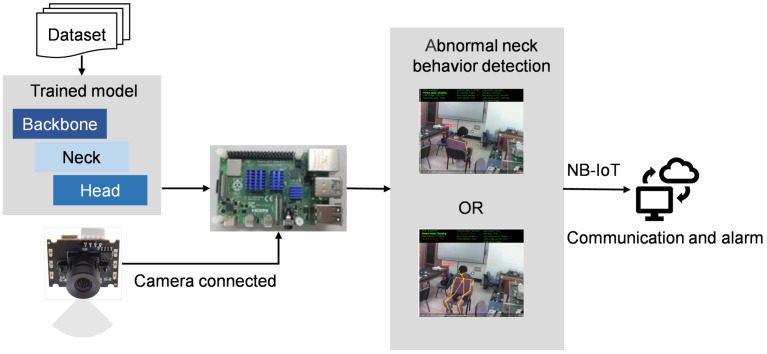
NABNet-based IoT alert system structure.

We constructed a NABNet-based IoT alert system for abnormal behavior detection to identify head tilt and dropped head postures. The structure of the system is illustrated in Figure 6. The edge device must process the video the camera collects to track the object and determine the abnormal behavior, which requires matching computational power. The selected edge devices must also be extensible to enhance the system’s availability. We chose the Raspberry Pi 3B as the control terminal. The Raspberry Pi 3B is known for its compact size and powerful functionality and is widely used in fields such as smart homes, media devices, and industrial control. Its primary parameters are detailed in Table 1. We selected the DF200 camera, which has a resolution of 1280 × 1080P and a speed of 30 frames per second.

The system captures indoor video through camera monitoring and transmits it to edge devices for analysis using NABNet. The edge devices continue to track objects even when no abnormal behavior events are detected. In the event of a detection, the system interacts with the cloud and issues an alert.

## 5. Experiments

In this section, we introduce the experiment methodology and evaluate the performance of NABNet. We also carry out a series of ablation studies.

### 5.1. Setup

**Figure 7:**
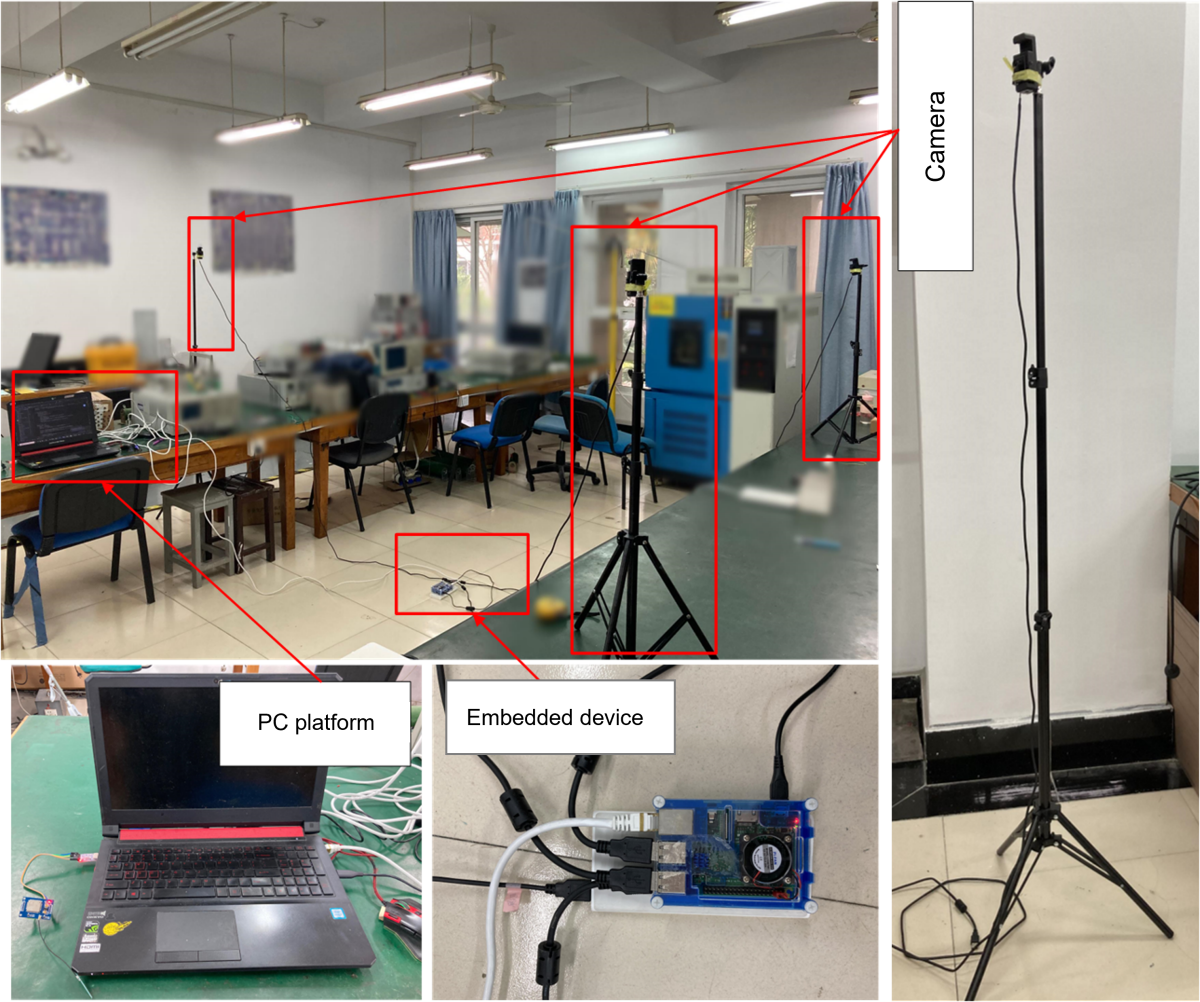
Experimental setup.

We conducted field experiments to evaluate the effectiveness of our approach, consisting of a camera and 1.7 m bracket, embedded devices, and a computer, as shown in Figure 7. We selected 80 participants, including 60 males and 20 females, aged between 24 and 30 years old and varying in height between 1.55 m and 1.85 m. The participants performed positive pose (head tilt or dropped head) and negative pose (front position) at distances of 0.5 m and 0.75 m from the camera.

The performance of the proposed model was evaluated using the Precision, Recall, and Accuracy:

(5) $$Precision=\frac{TP}{TP+FP}$$

(6) $$Recall=\frac{TP}{TP+FN}$$

(7) $$Accuracy=\frac{TP+TN}{TP+FP+TN+FN}$$

where TP is the number of positive samples correctly detected, FN is the number of negative samples incorrectly detected, FP is the number of positive samples falsely classified as negative ones, and TN is the number of negative samples correctly detected.

### 5.2. Evaluation of NABNet-Based IoT System

**Figure 8:**
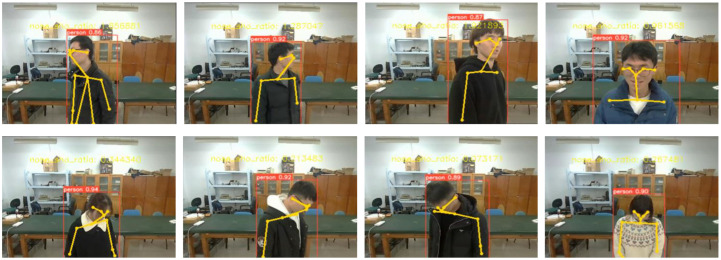
Representative images of positive and negative poses.

The participants performed positive and negative poses facing the camera in the front position, 30° right rotation, 30° left rotation, 60° right rotation, and 60° left rotation, as shown in Figure 8. The sample number was 160 (80 persons × two poses). The experimental results in Table 2 show that the detection accuracy in the front position and at 30° rotation is higher than that at 60° rotation. In general, as the rotation angle increases, the detection accuracy gradually decreases.

**Figure 9:**
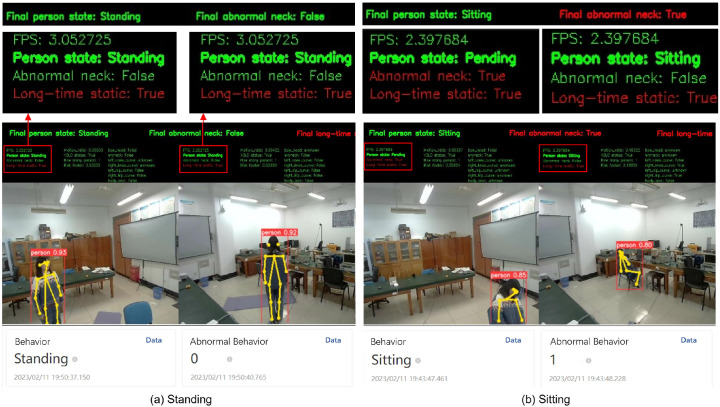
Illustration of abnormal neck behavior detection displayed on the server screens and cloud.

Figure 9 shows the abnormal behavior neck detection results obtained with our multi-node system; (a) shows results for the control group when the participant is standing and is detected correctly by the system, while (b) shows the results when the participants are partially occluded. In the latter case, the system is still able to detect abnormal behavior of the neck. The multi-node detection and final results are shown above the monitoring video, with the final result comprising a comprehensive judgment of the multi-node detection results. The remote monitoring data on the cloud platform are shown below the monitoring video, indicating whether an abnormal behavior event occurs and an alarm was realized.

### 5.3. Ablation Studies

Ablation experiments were conducted on the different improvement stages of NABNet, including the CA mechanism, tracker, and angle correction. We selected five groups of experimental fragments from the Multicam Fall Database [43], Le2i [44], SIMPLE Fall Detection Dataset [45], and one group photographed in a low illumination scene.

Table 3 shows the influence of the different components on our model. It can be observed that incorporating the CA mechanism leads to a 3.58% improvement in accuracy, indicating that adding the CA mechanism enhances the model’s accuracy in detecting small targets.

To verify the effectiveness of the proposed tracker, we compared it with Kernelized Correlation Filters (KCF) [46] and Tracking Learning Detection (TLD) [47]. Table 3 shows that the accuracy of our tracker is on par with KCF, while the accuracy of TLD is significantly lower, indicating that TLD incurs errors in object tracking. In order to better meet the system’s real-time performance requirements, we tested the frame rates of three different trackers, with the results presented in Table 4. Our tracker achieved the highest frame rate, with KCF being 25.57 frames per second (fps) slower. It demonstrates that our algorithm offers superior detection speed and improved real-time capabilities while maintaining comparable accuracy.

By utilizing angle correction via affine transformation, our method can correct the object to the forward position when facing the camera at an angle to obtain the actual neck angle information. As shown in the last two lines of Table 3, the accuracy of the model is improved by 8.72% after affine transformation.

## 6. Conclusions

Focusing on the local behavior of abnormal neck postures, this paper proposes an abnormal behavior detection network called NABNet based on object detection and pose estimation. To accommodate devices with limited computing power, we have also developed an IoT detection and alert system for edge devices based on NABNet, which can realize monitoring and early alerts on a cloud platform. Our experimental results show the effectiveness of the proposed approach.

As with the majority of studies, the design of the current study is subject to limitations. We have not yet validated the effectiveness of our approach on a large-scale public dataset specifically for neck abnormal behavior due to the absence of such a dataset, which highlights the need for further research in this area. In the future, we will consider increasing the number of key points and combining NABNet with other pose estimation models to improve its ability to detect multiple abnormal behaviors.

## Figures and Tables

**Table 1:** Primary parameters of the Raspberry Pi 3B.

Parameters	Configuration
SOC	CM2711
CPU	ARM Cortex-A72 1.5 GHz
GPU	Broadcom VideoCore IV
Memory	4 GB LPDDR4
Power	5 V Micro USB
Supported Systems	Raspbian/Ubuntu/Windows10/Linux

**Table 2:** Evaluation of our system based on the obtained samples.

	0.5 m			0.75 m		
**Position**	**Recall (%)**	**Precision (%)**	**Accuracy (%)**	**Recall (%)**	**Precision (%)**	**Accuracy (%)**
Front position	97.50	96.30	96.88	96.25	96.25	96.25
30° right rotation	97.50	98.73	98.13	95.00	97.44	96.25
30° left rotation	95.00	97.44	96.25	97.50	93.98	95.63
60° right rotation	86.25	92.00	89.38	86.25	87.34	86.88
60° left rotation	88.75	91.03	90.00	87.50	88.61	88.13
Mean	93	95.1	94.13	92.5	92.72	92.63

**Table 3:** Influence of components on our model.

Model	CA Mechanism	Tracker	Angle Correction	Accuracy (%)
YOLOv5s				67.44
YOLOv5s	*√*			71.02
YOLOv5s	*√*	KCF		87.45
YOLOv5s	*√*	TLD		78.63
YOLOv5s	*√*	Ours		85.41
YOLOv5s	*√*	Ours	*√*	94.13

**Table 4:** Comparison experiments of the detection frame rate with different trackers.

Tracker	Detection Frame Rate (fps)
KCF	17.61
TLD	9.95
Ours	43.18

## Data Availability

The datasets generated and analyzed during the current study are available from the corresponding author upon reasonable request.

## References

[1] Doewes R.I., Gharibian G., Zaman B.A., Akhavan-Sigari R. (2023). An updated systematic review on the effects of aerobic exercise on human blood lipid profile. Curr. Probl. Cardiol..

[2] Wang L., Zhou Y., Li R., Ding L. (2022). A fusion of a deep neural network and a hidden Markov model to recognize the multiclass abnormal behavior of elderly people. Knowledge-Based Syst..

[3] Liu C., Zhang Y., Xue Y., Qian X. (2023). AJENet: Adaptive joints enhancement network for abnormal behavior detection in office scenario. IEEE Trans. Circuits Syst. Video Technol..

[4] Ma C., Du J., Gravina R. (2022). Abnormal behavior detection based on activity level using fuzzy inference system for wheelchair users. Human-Centric Comput. Inf. Sci..

[5] Arifoglu D., Wang Y., Bouchachia A. (2021). Detection of dementia-related abnormal behaviour using recursive auto-encoders. Sensors.

[6] Tokas P. (2023). Machine learning based text neck syndrome detection using Microsoft Kinect sensor. Mater. Today Proc..

[7] Alruwaili M., Siddiqi M.H., Atta M.N., Arif M. (2024). Deep learning and ubiquitous systems for disabled people detection using YOLO models. Comput. Hum. Behav..

[8] Fu Y., Ran T., Xiao W., Yuan L., Zhao J., He L., Mei J. (2024). GD-YOLO: An improved convolutional neural network architecture for real-time detection of smoking and phone use behaviors. Digit. Signal Process..

[9] Cao C., Lan C., Zhang Y., Zeng W., Lu H., Zhang Y. (2018). Skeleton-based action recognition with gated convolutional neural networks. IEEE Trans. Circuits Syst. Video Technol..

[10] Lentzas A., Vrakas D. (2020). Non-intrusive human activity recognition and abnormal behavior detection on elderly people: A review. Artif. Intell. Rev..

[11] YOLOv5. https://github.com/ultralytics/yolov5.

[12] Hou Q., Zhou D., Feng J. Coordinate attention for efficient mobile network design. Proceedings of the IEEE/CVF Conference on Computer Vision and Pattern Recognition.

[13] Osokin D. (2018). Real-time 2d multi-person pose estimation on cpu: Lightweight openpose. arXiv.

[14] Zhang D., Han J., Cheng G., Yang M.H. (2021). Weakly supervised object localization and detection: A survey. IEEE Trans. Pattern Anal. Mach. Intell..

[15] Chaoxia C., Shang W., Zhang F., Cong S. (2022). Weakly aligned multimodal flame detection for fire-fighting robots. IEEE IEEE Trans. Ind. Inform..

[16] Girshick R., Donahue J., Darrell T., Malik J. Rich feature hierarchies for accurate object detection and semantic segmentation. Proceedings of the IEEE Conference on Computer Vision and Pattern Recognition.

[17] Ren S., He K., Girshick R., Sun J. (2015). Faster r-cnn: Towards real-time object detection with region proposal networks. Adv. Neural Inf. Process. Syst..

[18] Dai J., Li Y., He K., Sun J. (2016). R-fcn: Object detection via region-based fully convolutional networks. Neural Inf. Process. Syst..

[19] Pang J., Chen K., Shi J., Feng H., Ouyang W., Lin D. Libra r-cnn: Towards balanced learning for object detection. Proceedings of the IEEE/CVF Conference on Computer Vision and Pattern Recognition.

[20] Chen Z., Chen D., Zhang Y., Cheng X., Zhang M., Wu C. (2020). Deep learning for autonomous ship-oriented small ship detection. Saf. Sci..

[21] Redmon J., Divvala S., Girshick R., Farhadi A. You only look once: Unified, real-time object detection. Proceedings of the IEEE Conference on Computer Vision and Pattern Recognition.

[22] Liu W., Anguelov D., Erhan D., Szegedy C., Reed S., Fu C.Y., Berg A.C. Ssd: Single shot multibox detector. Proceedings of the Computer Vision—ECCV 2016: 14th European Conference.

[23] Lin T.Y., Goyal P., Girshick R., He K., Dollár P. Focal loss for dense object detection. Proceedings of the IEEE International Conference on Computer Vision.

[24] Li Y., Xue Y., Li L., Zhang X., Qian X. (2022). Domain adaptive box-supervised instance segmentation network for mitosis detection. IEEE Trans. Med. Imag..

[25] Li X., Lai S., Qian X. (2021). Dbcface: Towards pure convolutional neural network face detection. IEEE Trans. Circuits Syst. Video Technol..

[26] Liu C., Da Z., Liang Y., Xue Y., Zhao G., Qian X. (2022). Product recognition for unmanned vending machines. IEEE Trans. Neural Netw. Learn. Syst..

[27] Li H., Wu D., Zhang W., Xiao C. (2023). YOLO-PL: Helmet wearing detection algorithm based on improved YOLOv4. Digit. Signal Process..

[28] Qiu J., Yan X., Wang W., Wei W., Fang K. (2021). Skeleton-based abnormal behavior detection using secure partitioned convolutional neural network model. IEEE J. Biomed. Health Inform..

[29] Naser A., Lotfi A., Mwanje M.D., Zhong J. (2022). Privacy-preserving, thermal vision with human in the loop fall detection alert system. IEEE T. Hum.-Mach. Syst..

[30] Jin F., Zhang R., Sengupta A., Cao S., Hariri S., Agarwal N.K., Agarwal S.K. Multiple patients behavior detection in real-time using mmWave radar and deep CNNs. Proceedings of the 2019 IEEE Radar Conference.

[31] Okumura N., Yamanoi Y., Kato R., Yamamura O. Fall detection and walking estimation using floor vibration for solitary elderly people. Proceedings of the 2019 IEEE International Conference on Systems, Man and Cybernetics (SMC).

[32] Santos G.L., Endo P.T., Monteiro K.H.d.C., Rocha E.d.S., Silva I., Lynn T. (2019). Accelerometer-based human fall detection using convolutional neural networks. Sensors.

[33] Alruwaili M., Atta M.N., Siddiqi M.H., Khan A., Khan A., Alhwaiti Y., Alanazi S. (2023). Deep Learning-Based YOLO Models for the Detection of People With Disabilities. IEEE Access.

[34] Fang M.-T., Chen Z.-J., Przystupa K., Li T., Majka M., Kochan O. (2021). Examination of abnormal behavior detection based on improved YOLOv3. Electronics.

[35] Mehmood A. (2021). Lightanomalynet: A lightweight framework for efficient abnormal behavior detection. Sensors.

[36] Bochkovskiy A., Wang C., Liao H. (2020). Yolov4: Optimal speed and accuracy of object detection. arXiv.

[37] Gündüz M., Işık G. (2023). A new YOLO-based method for real-time crowd detection from video and performance analysis of YOLO models. J. Real-Time Image Process..

[38] Rezatofighi H., Tsoi N., Gwak J., Sadeghian A., Reid I., Savarese S. Generalized intersection over union: A metric and a loss for bounding box regression. Proceedings of the IEEE/CVF Conference on Computer Vision and Pattern Recognitio.

[39] Dai Y., Yu S., Yan Y. (2019). An adaptive EKF-FMPC for the trajectory tracking of UVMS. IEEE J. Ocean. Eng..

[40] Maji D., Nagori S., Mathew M., Poddar D. Yolo-pose: Enhancing yolo for multi person pose estimation using object keypoint similarity loss. Proceedings of the IEEE/CVF Conference on Computer Vision and Pattern Recognition.

[41] Cao Z., Simon T., Wei S.E., Sheikh Y. Realtime multi-person 2d pose estimation using part affinity fields. Proceedings of the IEEE Conference on Computer Vision and Pattern Recognition.

[42] Stearns C., Kannappan K. (1995). Method for 2-D Affine Transformation of Images. US Patent.

[43] Auvinet E., Rougier C., Meunier J., St-Arnaud A., Rousseau J. (2010). Multiple cameras fall dataset. DIRO-Université Montréal Tech. Rep..

[44] Charfi I., Miteran J., Dubois J., Atri M., Tourki R. (2013). Optimized spatio-temporal descriptors for real-time fall detection: Comparison of support vector machine and Adaboost-based classification. J. Electron. Imag..

[45] Chua J.-L., Chang Y.C., Lim W.K. (2015). A simple vision-based fall detection technique for indoor video surveillance. Signal Image Video Process..

[46] Henriques J.F., Caseiro R., Martins P., Batista J. (2014). High-speed tracking with kernelized correlation filters. IEEE Trans. Pattern Anal. Mach. Intell..

[47] Kalal Z., Mikolajczyk K., Matas J. (2011). Tracking-learning-detection. IEEE Trans. Pattern Anal. Mach. Intell..

